# Use of a Pollen-Based Diet to Expose the Ladybird Beetle *Propylea japonica* to Insecticidal Proteins

**DOI:** 10.1371/journal.pone.0085395

**Published:** 2014-01-07

**Authors:** Xiaojie Zhang, Yunhe Li, Jörg Romeis, Xinming Yin, Kongming Wu, Yufa Peng

**Affiliations:** 1 College of Life Science, Henan Agricultural University, Zhengzhou, China; 2 State Key Laboratory for Biology of Plant Diseases and Insect Pests, Institute of Plant Protection, Chinese Academy of Agricultural Sciences, Beijing, China; 3 Agroscope, Institute for Sustainability Sciences ISS, Zurich, Switzerland; University of Tennessee, United States of America

## Abstract

A rape seed pollen-based diet was developed and found to be suitable for use in a dietary exposure assay for *Propylea japonica*. Using the diet, we established and validated a dietary exposure assay by using the protease inhibitor E-64 as positive control. Dose-dependent responses were documented for all observed life-table parameters of *P. japonica* including survival, pupation and eclosion rates, development time and adult weight. Results suggested that the dietary assay can detect the effects of insecticidal compounds on the survival and development of *P. japonica*. Using the established dietary assay, we subsequently tested the toxicity of Cry1Ab, Cry1Ac and Cry1F proteins that are expressed by transgenic maize, cotton or rice plants to *P. japonica* larvae. The diet containing E-64 was included as a positive control. Survival and development of *P. japonica* larvae were not adversely affected when the diet contained purified Cry1Ab, Cry1Ac, or Cry1F at 500 µg/g diet representing a worst-case exposure scenario. In contrast, *P. japonica* larvae were adversely affected when the diet contained E-64. The bioactivity and stability of the Cry proteins in the diet and Cry protein uptake by the ladybird larvae were confirmed by bioassay with a Cry-sensitive insect species and by ELISA. The current study describes a suitable experimental system for assessing the potential effects of gut-active insecticidal compounds on ladybird beetle larvae. The experiments with the Cry proteins demonstrate that *P. japonica* larvae are not sensitive to Cry1Ab, Cry1Ac and Cry1F.

## Introduction

Before commercial cultivation, a new genetically engineered (GE) plant variety has to pass a rigorous environmental risk assessment. An important component of this assessment is the evaluation of potential adverse effects on valued non-target organisms (NTOs), which is especially relevant for GE plants that express novel insecticidal genes [1]–[3]. The non-target risk assessment follows a tiered framework that typically starts with laboratory toxicity studies that are referred to as Tier-1 assays and are conducted under controlled, worst-case exposure conditions [1], [4], [5]. The objective of these studies was to identify the potential toxicity of the insecticidal proteins produced by the insect-resistant GE (IRGE) plants on surrogate species, i.e., on species that are representative of valued NTOs in the environment in which the IRGM crop is going to be released [1], [4]–[7].

Ladybird beetles (Coleoptera: Coccinellidae) are well-known predators of Sternorrhyncha (Hemiptera), such as aphids, scale insects, psyllids, and white flies, but also prey on a variety of other soft-bodied arthropods. They thus play an important role in biological pest control in multiple crop systems including cotton, maize, and rice [8], [9]. Therefore this group of insect predators has been in the focus of risk assessment studies with IRGE plants [10]–[26].

To assess the direct toxicity of insecticidal proteins on ladybird beetles, dietary exposure assays have been developed for the species *Coleomegilla maculata, Coccinella septempunctata*, and *Adalia bipunctata*, which are all easily available and suitable for laboratory testing [27]. Among the three species, *C. maculata* is particularly suitable as a test organism because it can be exposed to high doses of a test compound mixed into a shrimp-egg-based diet [18], [19] or in form of GE plant pollen [10]. However the dietary system can not be used in China since there is no *C. maculata* available in Chinese agricultural ecosystems.

*Propylea japonica* (Coleoptera: Coccinellidae) is a very common and abundant predator throughout East Asia in many crop systems including maize, cotton, rice, vegetables, and fruit trees [12], [28]–[30]. Both larvae and adults of *P. japonica* are predacious and feed preferably on aphids, planthoppers and whiteflies [31]. In addition, they are known to use plant pollen as a supplemental food source [32], [33]. Similar to *C. maculata*, the beetles can be directly exposed to plant-produced insecticidal proteins when foraging pollen in IRGE crops. For example, nearly 400 ng Cry2Aa per g dry weight (DW) were detected in adult *P. japonica* by Enzyme-Linked Immunosorbent Assays (ELISA) when they were collected in *Bt* rice during anthesis, while no Cry2Aa protein was detected when beetles were collected in the same field after anthesis [33].

For the current report, we developed an experimental system for evaluating the direct effects of insecticidal compounds on *P. japonica*. The experimental system was subsequently used to assess the effects of Cry1Ab, Cry1Ac, and Cry1F on *P. japonica* larvae. These proteins are produced by several GE crops including cotton, maize and rice.

## Results

### Pollen Consumption of *P. japonica*

To establish a rape seed (*Brassica napus* L.) pollen-based diet that can be used to provide high doses of insecticidal compounds to *P. japonica*, we measured whether the larvae readily accept the rape seed pollen and how many pollen grains were consumed. Rape seed pollen was provided on the first day of each instar, then a mixture of pollen and soybean aphids were provided until development into the next instar.

**Figure 1:**
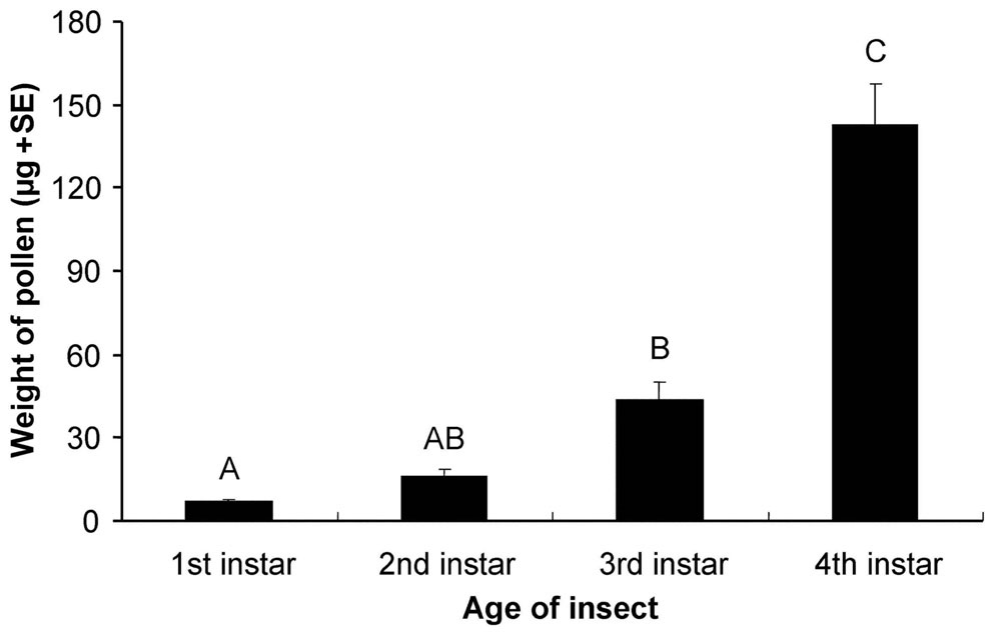
Consumption of rape seed pollen by *Propylea japonica* larvae. Larvae were fed exclusively with pollen for one day and the amount of pollen consumed is shown as weight of pollen per insect gut (mean+SE). Different letters above bars indicate significant differences (Tukey HSD test) (n = 7–10).

The mean (± SE) DW of a single rape seed pollen grain was estimated to be 9.8±0.46 ng. As expected, the pollen consumption increased significantly with the growth of the *P. japonica* larvae (One-Way ANOVA: *F* = 49.9, *P*<0.001) and the calculated amount (weight) of pollen that was consumed by the different instars differed significantly and increased from first to fourth instar (Fig. 1).

### Fitness of *P. japonica* Feeding on Rape Seed Pollen

**Figure 2:**
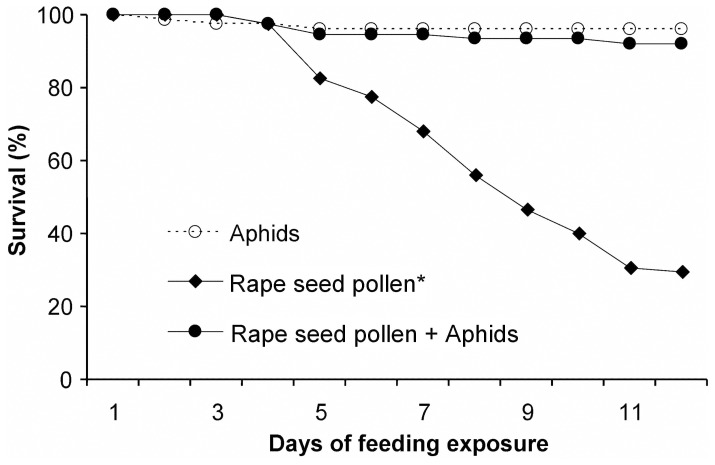
Survival of *Propylea japonica* larvae on different diets. Larvae were fed exclusively with aphids or pollen or a combination of the two. An asterisk denotes a significant difference to the other two treatments (n = 75).

The suitability of the pollen for sustaining normal survival and development of *P. japonica* was evaluated. When fed exclusively with soybean aphids or with a combination of pollen and aphids (as described above), over 92% of *P. japonica* larvae reached the pupal stage and over 90% developed to adults (Table 1). No significant difference was detected between these two treatments for the pupation and eclosion rates (*χ^2^* = 1.06, *P = *0.30 and *χ^2^* = 0.08, *P = *0.77, respectively) (Table 1), and for 12-day survival rates (*χ^2^* = 2.75, *P = *0.10) (Fig. 2). Likewise no significant difference was detected for the parameter of adult fresh weight (FW) (females: t = 0.93, *P* = 0.35; males: t = 0.65, *P* = 0.52) (Table 1). However the insects feeding on the pollen/aphid diet had a significantly longer larval development time compared to those feeding exclusively on aphids (U = 276.0, *P*<0.001). No difference was detected for pupal development time between the two treatments (U = 2090.5, *P* = 0.27). In contrast, the total fecundity of the females feeding on the pollen/aphid diet was significantly higher than those feeding on aphids only (t = −2.68, df = 54, *P* = 0.01) (Table 1). In the pollen only treatment, few insects reached the pupal stage and the larval developmental time was significantly longer than for those feeding solely on soybean aphids or pollen-based diets (both P<0.001) (Table 1, Fig. 2).

**Table 1:** Performance of *Propylea japonica* on different diets.

Parameter	Soybean aphids	Rape seed pollen/soybean aphids	Rape seed pollen
Pupation rate (%)^a^	96.00 (75) a	92.00 (75) a	6.67 (75) b
Eclosion rate (%)^a^	90.67 (75) a	92.00 (75) a	–
Larval development time^b^	6.39±0.11 (68) a	8.07±0.14 (69) b	12.6±0.40 (5) c
Pupal development time^b^	3.76±0.04 (68) a	3.82±0.04 (69) a	–
Female fresh weight (mg)^c^	7.34±0.13 (39) a	7.16±0.15 (29) a	–
Male fresh weight (mg)^c^	5.99±0.12 (36) a	5.87±0.14 (33) a	–
Total fecundity^c^	64.86±5.85 (28) a	93.18±8.80 (28) b	–

Life-table parameters (± SE) of *P. japonica* larvae when fed exclusively with aphids or pollen or when fed pollen during the first day of each larval staged and then subsequently received pollen and soybean aphids until development into the next instar. Number of replicates is given in parentheses. Different letters following means in the same row denote significant difference between treatments.

^a^χ^2^ test (pupation rate: Bonferroni correction lead to adjusted α = 0.017).

b Mann-Whitney U-test (larval development time: Bonferroni correction lead to adjusted α = 0.017).

c Student’s t-test.

### Validation of the Dietary Test System

**Figure 3:**
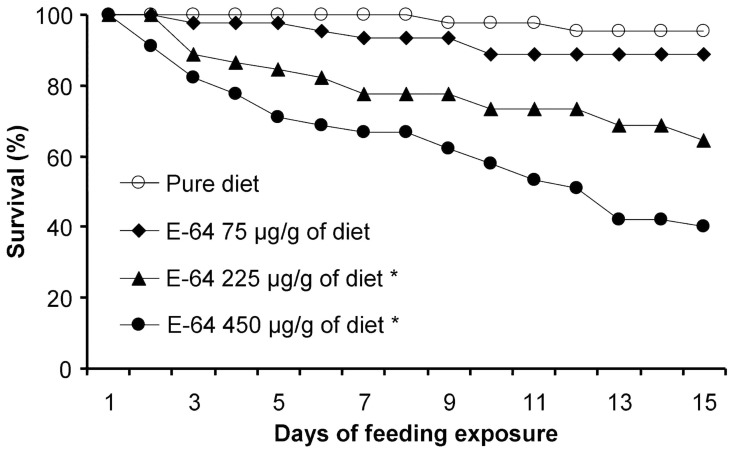
Survival of *Propylea japonica* larvae when fed different concentrations of E-64. Larvae were fed a combination of rape seed pollen into which the E-64 was incorporated and soybean aphids. An asterisk denotes a significant difference between an E-64 treatment and the control (n = 45).

To validate the test system, experiments were conducted using the protease inhibitor E-64 as the test compound. In the untreated control treatment, over 95% of *P. japonica* larvae developed to adults (Table 2). With increasing concentration of E-64 in the diet, the survival rates of *P. japonica* were steadily reduced (Table 2, Fig. 3). While survival analysis revealed no statistical difference between the treatment containing E-64 at 75 µg/g diet and the control (*χ^2^* = 1.44, *P = *0.23), survival rates were significantly decreased compared to the control for insects fed diets containing E-64 at 225 and 450 µg/g diet (both *P*<0.001) (Fig. 3). A similar dose-dependent response was found for the other life-table parameters recorded (Table 3).

**Table 2:** Performance of *Propylea japonica* when fed different concentrations of E-64.

Treatment	Control (pure diet)	E-64 (75 µg/g diet)	E-64 (225 µg/g diet)	E-64 (450 µg/g diet)
Pupation rate (%)^a^	97.78 (45)	91.11 (45)	60.00 (45)*	44.44 (45)*
Eclosion rate (%)^a^	95.56 (45)	88.89 (45)*	37.78 (45)*	35.56 (45)*
Days to adult^b^	13.29±0.10 (43)	14.20±0.09 (40)*	14.21±0.13 (17)*	14.94±0.28 (16)*
Female fresh weight (mg)^c^	7.07±0.16 (23)	7.14±0.16 (22)	5.76±0.18 (10)*	5.21±0.31 (8)*
Male fresh weight (mg)^c^	5.90±0.17 (20)	6.10±0.18 (18)	4.82±0.08 (7)*	4.74±0.24 (8)*

Life-table parameters (± SE) of *P. japonica* larvae when fed rape seed pollen containing different concentrations of E-64. Number of replicates is given in parentheses. Insects were fed exclusively with pollen for the first day of each larval stage and then subsequently received pollen and soybean aphids until development into the next instar. Each E-64 treatment was compared to the control. An asterisk denotes a significant difference between an E-64 treatment and the control (P<0.05).

a χ^2^ test with Bonferroni correction (adjusted α = 0.017).

b Mann-Whitney U–test with Bonferroni correction (adjusted α = 0.017).

c Dunnett test.

**Table 3:** Effects of insecticidal proteins on *Propylea japonica.*

Treatment	Pupation rate (%)^a^	Eclosion rate (%)^a^	Days to pupa (d)^b^	Adult fresh weight (mg)^c^	
				Female	Male
Untreated control	82.22 (45)	82.22 (45)	8.55±0.14 (37)	6.47±0.19 (20)	5.42±0.12 (17)
Cry1Ab	86.67 (45)	82.22 (45)	8.85±0.14 (39)	6.79±0.18 (16)	5.71±0.15 (21)
Cry1Ac	80.00 (45)	80.00 (45)	8.71±0.14 (36)	6.90±0.11 (20)	5.70±0.18 (15)
Cry1F	86.67 (45)	84.44 (45)	8.67±0.15 (39)	6.96±0.22 (17)	5.79±0.14 (21)
E-64	55.56 (45)*	24.44 (45)*	11.56±0.22 (25)*	5.96±0.39 (6)	5.40±0.23 (5)

Life-table parameters (± SE) of *P. japonica* larvae when fed rape seed pollen containing Cry1Ab, Cry1Ac, Cry1F (500 µg/g pollen) or E-64 (400 µg/g pollen). Number of replicates is given in parentheses. Insects were fed exclusively with pollen for the first day of each larval stage and then subsequently received pollen and soybean aphids until development into the next instar. Each toxin treatment was compared to the control. An asterisk denotes a significant difference between a toxin treatment and the control.

a χ^2^ test with Bonferroni correction (adjusted α = 0.013).

b Mann-Whitney U-test with Bonferroni correction (adjusted α = 0.013).

c Dunnett test.

### Toxicity of Cry Proteins to Larvae of *P. japonica*

#### Effects on life-table parameters

**Figure 4:**
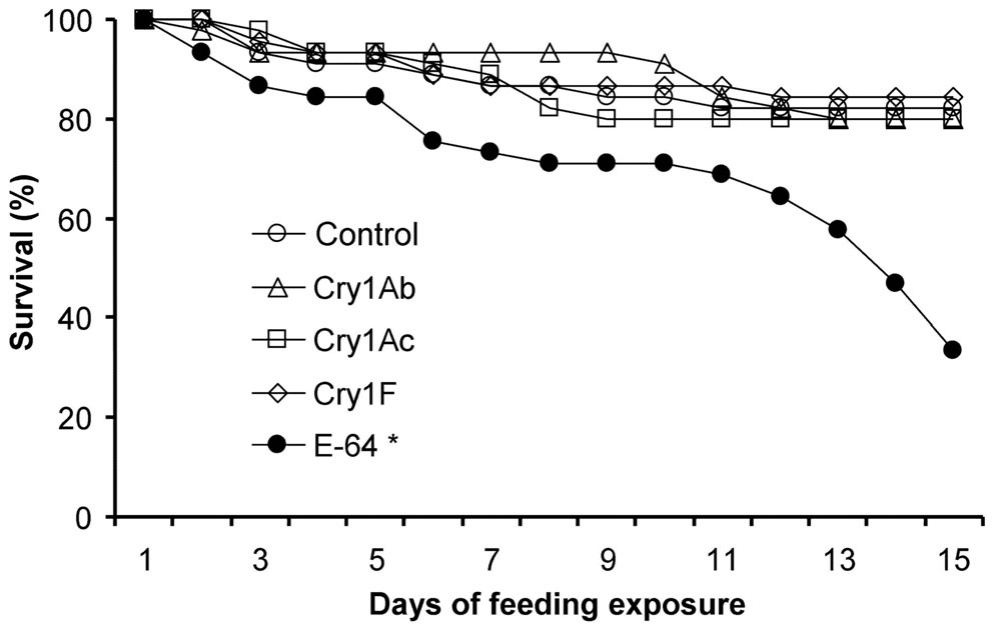
Survival of *Propylea japonica* larvae when pollen left untreated or containing different insecticidal proteins. Larvae were fed a combination of rape seed pollen into which the insecticidal proteins were incorporated and soybean aphids. An asterisk denotes a significant difference between an insecticidal protein and the control (n = 45).

The dietary toxicity assay developed here was used to assess the toxicity of Cry1Ab, Cry1Ac, and Cry1F to *P. japonica* larvae. Pair-wise comparisons revealed that the treatments containing Cry1Ab, Cry1Ac, or Cry1F protein did not differ significantly from the untreated (negative) control for the parameters of pupation rate (Cry1Ab: *χ^2^* = 0.34, *P* = 0.56; Cry1Ac: *χ^2^* = 0.07, *P* = 0.79; Cry1F: *χ^2^* = 0.33, *P* = 0.56), eclosion rate (Cry1Ab: *χ^2^* = 0.00, *P* = 0.61; Cry1Ac: *χ^2^* = 0.07, *P* = 0.50; Cry1F: *χ^2^* = 0.08, *P* = 0.50), and larval development time (Cry1Ab: *U* = 546.50, *P* = 0.13; Cry1Ac: *U* = 582.50, *P* = 0.35; Cry1F: *U* = 656.50, *P* = 0.62) (Table 3). Similarly, no significant difference was detected between each of the Cry proteins and the negative control treatment for female and male FW (Dunnett’s test; all *P*>0.1). In contrast, insects fed E-64 had significantly decreased pupation (*χ^2^* = 7.47, *P* = 0.01) and eclosion rates (*χ^2^* = 30.18, *P*<0.001), and had a significantly longer larval developmental time (*U* = 10, *P*<0.001) than those in the negative control (Table 3). Weight of the emerging adults, however, was not affected by E-64 (*P*>0.05 for both sexes). The survival curves were significantly affected by diet (*χ^2^* = 43.32, df = 4, *P*<0.001). Pair-wise comparisons revealed no statistical differences between any Cry protein treatment and the control (*P*>0.50), while *P. japonica* survival was significantly lower in the E-64 treatment (*χ^2^* = 11.1, *P = *0.001) (Fig. 4).

#### Uptake of cry proteins by *P. japonica*

According to our measurements by double-antibody sandwich enzyme-linked immunosorbent assays (DAS-ELISA), all *P. japonica* larvae contained Cry1Ab, Cry1Ac, or Cry1F protein in the *Bt* treatments but none contained Cry protein in the control. The mean (± SE) concentrations of Cry1Ab, Cry1Ac, and Cry1F detected in 4^th^ instar larvae of *P. japonica* were 9.67±7.10, 10.79±5.02, and 11.17±3.28 µg/g DW of insects, respectively. No Cry protein was detected in *P. japonica* pupae.

#### Stability and bioactivity of cry proteins

The detection rates of the ELISA for the three Cry proteins after mixing into the rape seed pollen was approximately 56–83% of the nominal concentrations; the mean (± SE) concentrations (µg/g FW of pollen) detected were 417.4±26.8 for Cry1Ab, 402.2±10.4 for Cry1Ac, and 278.5±19.1 for Cry1F (n = 5). After the 2-day feeding period, the mean Cry protein concentration in the diet had declined by 5.7 to 23.6%. This decline was significant for Cry1F (212.7±8.7 µg/g; Student’s t-test; *t* = 3.14, *df = *4, *P = *0.04) but not for Cry1Ac (379.2±19.9 µg/g; *t* = 1.02, *df = *4, *P = *0.37) and Cry1Ab (447.9±33.8 µg/g; *t* = 0.66, *df = *3, *P* = 0.56).

**Figure 5:**
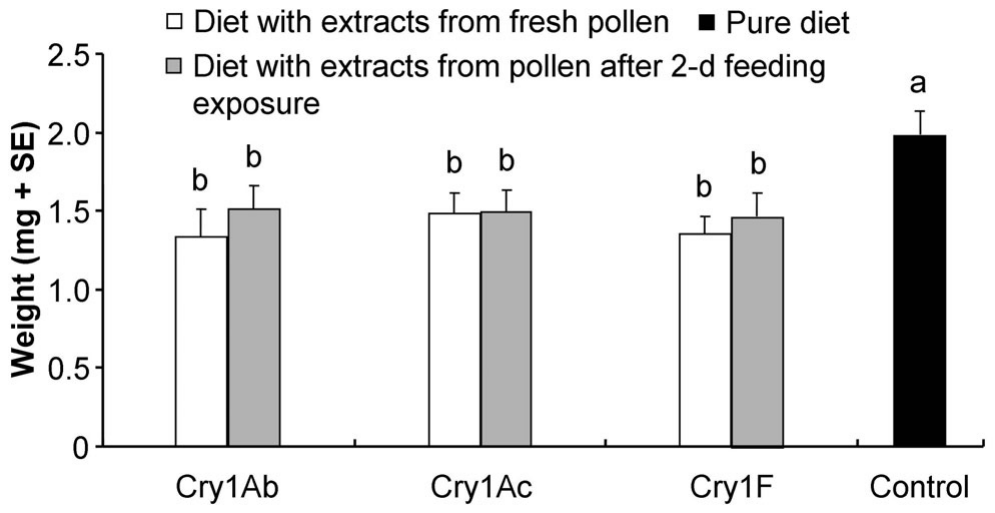
Sensitive insect bioassay with Cry protein treated rape seed pollen. Weight (mean+SE) of *Chilo suppressalis* larvae fed on artificial diets incorporated with exacts either from untreated rape seed pollen (control) or pollen containing Cry1Ab, Cry1Ac or Cry1F that had been freshly prepared or had been exposed to *Propylea japonica* larvae for 2 d (n = 30).

Sensitive-insect bioassays showed that the mean weight (± SE) of *C. suppressalis* larvae was 1.97±0.15 mg when fed an artificial diet incorporated with the extract from the pollen that had not been treated with Cry protein (control) for 7 d, which was significantly greater than those fed any diet containing Cry protein (all P<0.05) (Fig. 5). Pair-wise comparisons revealed no statistical differences between the weights of *C. suppressalis* larvae fed extract from Cry protein treated pollen that had been either freshly prepared or had been exposed to *P. japonica* larvae for 2 d (Fig. 5).

## Discussion

We have established the use of rape seed pollen to expose *P. japonica* to high doses of insecticidal compounds in laboratory toxicity assays. Rape seed pollen was readily accepted and utilized by *P. japonica*. With aging of the *P. japonica* larvae, the consumption of pollen was steadily increased, and at the fourth instar a single larva contained an average of more than 15,000 pollen grains (equivalent to about 150 µg DW). The results from our fitness bioassay indicated that a feeding regime where larvae were fed pollen only for the first day of each larval stage and subsequently received aphids in addition to the pollen, provided a highly suitable diet. More than 90% of the larvae survived to the adult stage and adult weight was similar to those fed exclusively with soybean aphids, which is the preferred natural prey. Interestingly, *P. japonica* fecundity increased by more than 40% when fed the pollen-aphid diet as compared to females that had only received aphid prey. Although the larval development of *P. japonica* was found to be significantly delayed when fed on pollen-based diet, it may not necessarily affect the suitability of the diet used in a dietary toxicity assay.

Feeding experiments with the protease inhibitor E-64 showed that the reported dietary exposure assay can efficiently detect toxic effects of a test compound. Dose-dependent responses were observed for important life-table parameters, including survival rate, pupation rate, eclosion rate, larval development time, and adult weight. The test system developed in the current study was capable of detecting dietary effects of the insecticidal compound, and larval survival and larval development time were found to be the most sensitive indicator of toxicity.

Using this test system, we assessed the potential toxicity of purified Cry1Ab, Cry1Ac, and Cry1F to *P. japonica* larvae. These Cry proteins have been widely used in transgenic varieties of various crops, including cotton, maize, and rice [34]. Our results revealed no detrimental impact of the tested Cry proteins on any of the *P. japonica* life-table parameters measured. Feeding on diets containing E-64, in contrast, significantly reduced the survival and development of *P. japonica* larvae. This positive control demonstrated that the test compounds were actually ingested and that our experimental system was able to detect adverse effects caused by toxic compounds in the diet.

When the hazard of an insecticidal compound to an organism is measured in a dietary exposure assay, the concentration, stability, and bioactivity of the test compound in the food source and the uptake of the compound by the test organisms need to be confirmed [6], [35]. In our study, the concentration of Cry protein mixed with the rape seed pollen was 500 µg/g FW, which was >>100 times higher than the content of Cry protein in pollen from *Bt* maize [36], *Bt* cotton [19], [37] and *Bt* rice [38]. In *Bt* crop fields, *P. japonica* would only be exposed to GE pollen during the pollen shedding period [12], [33]. Before and after pollen shedding, *P. japonica* mainly feed on aphids that likely contain only traces or no amount of Cry protein [39]. This was confirmed in field studies where ELISA measurements detected much Cry2Aa in adult *P. japonica* when they were collected in Bt rice field during anthesis, but not in beetles that were collected in the same field after anthesis [33]. Our ELISA tests indicated that the Cry protein concentrations in the pollen diets were relatively stable with 5.7–23.6% degradation of Cry proteins observed during the 2-day feeding exposure. Despite this degradation, the detected Cry protein concentrations in the diets were still more than 250 µg/g FW of diet and thus still >>100 times higher than the concentration likely to be encountered by the beetles in the field. Consequently, the beetles in our bioassays were continuously exposed to Cry protein concentrations that were orders of magnitude higher than the concentrations that beetles are exposed to in the field.

Our ELISA analyses detected high concentrations of the Cry proteins in *P. japonica* larvae, but no Cry protein was detected in pupae. This result is consistent with the result from a previous study in which *Stethorus punctillum* (Coleoptera: Coccinellidae) was fed with *Bt* protein-contained spider mites [17]. Furthermore, to clarify whether Cry proteins retained their bioactivity during the 2-day feeding exposure, a sensitive-insect bioassay with *C. suppressalis* larvae was conducted. Larval weight was selected as the measurement endpoint rather than larval mortality because it has been reported to be a more sensitive and reliable parameter to analyze Cry protein effects on sensitive lepidopteran larvae [40], [41]. The results revealed that in any treatment with Cry proteins, the mean weight of *C. suppressalis* larvae was significantly decreased by 25–32% compared to those fed on the control diet. More importantly, the weight did not differ between larvae that were fed diets incorporated with Cry protein extracts from a freshly prepared pollen diet or from pollen that had been exposed to *P. japonica* larvae for 2 days. The results confirmed that the Cry proteins retained their bioactivity during the 2 day feeding duration in our bioassay. The data therefore demonstrate that the *P. japonica* larvae were exposed to high concentrations of bioactive Cry proteins during the duration of the bioassay. We thus conclude that *P. japonica* larvae are not sensitive to Cry1Ab, Cry1Ac, or Cry1F at concentrations that are much higher than those encountered in the field.

Due to the ecological importance of predacious coccinellids, a number of studies have been conducted to assess the potential toxicity of the Cry proteins used in our study to several ladybird beetle species. Our findings are in agreement with the majority of published evidence. Regulatory studies using *Hippodamia convergens* did not reveal any adverse effects of any of the three Cry proteins evaluated in our study [42]–[44]. The majority of peer-reviewed studies where ladybirds were directly fed purified proteins or exposed to proteins in tri-trophic assays using non-sensitive herbivores as prey, revealed no adverse effects of Cry1Ab to *A. bipunctata* [21], [23], *C. maculata* [45], *S. punctillum* [20], *Cryptolaemus montrouzieri* [23], Cry1Ac to *C. maculata* [19], *Cycloneda sanguinea* [26], and Cry1F to *C. maculata* [25]. Conflicting results were reported for Cry1Ab effects on *Cheilomenes sexmaculatus* [15], *C. maculata* [14] and *A. bipunctata* [16]. However, these studies appear to have suffered from flawed methodologies [46]– and, in the case of *A. bipunctata*, could not be confirmed in subsequent studies [21], [23]. Of particular interest is the comparison of our results to earlier studies conducted with *P. japonica*. No effect of Cry1Ac on the fitness of *P. japonica* was observed in a previous study, in which *P. japonica* were fed *Bt*-treated Cry1Ac-resistant *Helicoverpa armigera* (Lepidoptera: Noctuidae) larvae for two generations [50]. In addition, *P. japonica* was not negatively affected when feeding on aphids that were collected from Cry1Ab or Cry1Ac expressing Bt cotton [51], Cry1Ab-expressing rice pollen [12], or *Nilaparvata lugens* (Homoptera: Delphacidae), that had been reared on Bt rice plants [13].

In summary, our study results are consistent with the laboratory studies that have reported a lack of direct toxic effects of Cry1 proteins on ladybird beetles as well as with the results from many field studies conducted with *Bt* maize or cotton that have provided no evidence for direct adverse effects on ladybird beetles and other natural enemies [52], [53]. The study provides a sound dietary exposure system that can be used to study the direct toxic effects of orally-active insecticidal compounds on lethal and sublethal endpoints of larvae of the ladybird beetle *P. japonica*.

## Materials and Methods

### Ethics Statement

No specific permits were required for the described field studies. The rice fields from which *P. japonica* used in this study were originally collected were owned by the author’s institute (Institute of Plant Protection, Chinese Academy of Agricultural Sciences, CAAS). These field studies did not involve endangered or protected species.

### Experimental Conditions

Insects were reared and experiments were conducted in climate chambers at 26±1°C, 75±5% RH, and a 16∶8 h light:dark cycle.

### Insects

Specimens of *P. japonica* were collected at the experimental field station of the Institute of Plant Protection, CAAS, near Langfang city, Hebei Province, China (39.5°N, 116.7°E) in 2012. A colony was subsequently maintained in the laboratory without introduction of field-collected insects for over 4 generations. Both larvae and adults of *P. japonica* were reared on soybean seedlings infested with *Aphis glycines* (Homoptera: Aphididae). Newly hatched (<12 h after emergence) *P. japonica* larvae were used for the experiments.

A *Bt*-susceptible strain of *Chilo suppressalis* (Lepidoptera: Crambidae) was used to test the bioactivity of the Cry proteins. This strain has been maintained on an artificial diet for over 40 generations in the laboratory [54].

### Insecticidal Compounds and Pollen

Insecticidal compounds used this study included the protease inhibitor [E-64; N-[N-(L-3-trans-carboxyoxirane-2-carbony1)-L-leucyl]-agmatine], and the *Bt* proteins Cry1Ab, Cry1Ac, and Cry1F. E-64 was purchased from Sigma-Aldrich (St. Louis, MO), and the *Bt* proteins were purchased from Envirotest-China (agent for Envirologix Inc., Portland, Maine, USA; www.envirotest-china.com). The proteins were produced and purified at the Department of Biochemistry, Case Western Reserve University (USA) (contact person: Dr. Marianne Pusztai-Carey). The protoxins from *Bacillus thuringiensis* had been expressed as single-gene products in *Escherichia coli*. The *E. coli-*expressed protoxin inclusion bodies were then dissolved and trypsinized, and isolated and purified by ion exchange HPLC followed by desalting and lyophilizing the pure fractions. Purity was about 94–96%. Bioactivity of the Cry proteins was confirmed in sensitive bioassays in our laboratory using neonate larvae of *C. suppressalis* that were fed for 7 days with artificial diet containing a range of Cry protein concentrations. The EC_50_ (toxin concentration resulting in 50% weight reduction compared to the control) was estimated to be 26.1, 52.0, and 1604.0 ng/ml for Cry1Ab, Cry1Ac, and Cry1F, respectively.

Bee-collected rape seed pollen used in the experiments was purchased from China-Bee Science & Technology Development Co., Ltd (Beijing, China).

### Establishment of a Dietary Toxicity Assay

#### Pollen-based diet

*P. japonica* larvae were individually confined in Petri dishes (6.0 cm diameter, 1.5 cm height) and fed with rape seed pollen on the first day of each instar, then a mixture of pollen and soybean aphids (natural food) were provided until development into the next instar. For adults, single pairs of *P. japonica* were confined in the same Petri dishes and fed with pollen or with a combination of pollen and soybean aphids every alternate day. Several folded paper tapes (0.6 cm width, 10 cm length) were provided as oviposition substrates. The pollen was ground and directly sprinkled on the bottom of the Petri dishes, while the aphids were provided on 2-cm segments of heavily infested soybean seedlings. An open 1.5 ml centrifuge tube containing solidified 1% agar solution was added to each Petri dish as water source.

#### Pollen consumption of *P. japonica*

For each larval stage more than 10 larvae were collected and frozen at −20°C after feeding exclusively on pollen for one day. To count the pollen grains in the larval gut, insects were thawed and excised, and the whole alimentary canal was excised. Subsequently, the gut was transferred to a 1.5 ml microreaction tube containing 100 ml fuchsin acid solution. The red color stained the pollen grains and facilitated counting. After the gut was ruptured with a thin needle, the pollen suspension was mixed using a Vortex mixer. An aliquot (2.5 µl) of the suspension was transferred to a glass slide, and pollen grains (including full, partially digested, and empty ones) was counted with a microscope at 50×magnification. Three subsamples were counted for each insect gut, and the mean number of pollen grains was multiplied by 40 to estimate the total number contained in an insect. Seven to 10 insects were analyzed for each instar.

To calculate the weight of pollen that is contained in the larval gut of *P. japonica*, the mean weight of a single pollen grain was evaluated. Lyophilized fresh pollen (1.0 mg) was mixed with 1500 µl fuchsin acid solution. The solution was diluted 10-folds. The pollen grains were counted in each of five 2.5 µl aliquots of the suspension with a microscope at 50×magnification as described above. The mean number of pollen grains in the aliquots was multiplied by 6000 to obtain the number in the whole sample. Finally, the weight of each sample (1.0 mg) was divided by the number of grains to obtain the mean DW of an individual pollen grain. Five samples were measured. Based on the mean individual DW of pollen grains and the number of pollen grains found in the larval guts, the mean weight of pollen in the larval gut of *P. japonica* was calculated.

#### Fitness of *P. japonica* feeding on rape seed pollen

The suitability of the rape seed pollen for sustaining normal survival and development of *P. japonica* was evaluated in a bioassay with 3 diet treatments: i) continuous feeding with soybean aphids; ii) feeding with rape seed pollen for one day and then with a combination of pollen and soybean aphids until development into the next larval stage; iii) continuous feeding with rape seed pollen. Pollen and aphids were supplied as described above. One *P. japonica* neonate was randomly selected and added to each Petri dish and 75 insects were tested for each treatment. The neonates were provided with an *ad libitum* food supply by replacing the aphids every day and pollen every 2 days. Development and mortality of the *P. japonica* larvae were recorded twice per day (9∶00 am, 9∶00 pm).

When the insects had developed into adults, the freshly emerged adults were weighed on an electronic balance (CPA224S, Sartorius, Germany; d = 0.1 mg). Then the sex of freshly emerged adults was determined and they were randomly paired. A single pair was kept in a Petri dish containing folded paper tapes and provided with the same food that had been provided to the larval stage as described above. No adults emerged in the pollen only treatment. Twenty-eight pairs of *P. japonica* were tested in each of other two treatments. Adults were observed daily and the number eggs produced per day was recorded for 2 weeks.

#### Validation of the dietary test system

The protease inhibitor E-64 was selected to validate the test system because its toxicity to other ladybird beetles has been established in previous studies, i.e., *C. maculata* [18], [19] and *C. septempunctata* [22] and because preliminary experiments in our laboratory indicated that the compound does also affect *P. japonica*.

A stock solution of E-64 was diluted with distilled water and mixed with the pollen to obtain concentrations of 0, 75, 225, and 450 µg/g FW of pollen. As in the previous experiments, pollen was provided during the first day of each larval stage while insects were fed a combination of pollen and aphids until development into the next instar. The experimental system and the provision of food were the same as described in the previous section. The experiment was initiated with 45 insects per treatment. Development and mortality of the *P. japonica* larvae were recorded twice per day (9∶00 am, 9∶00 pm). The experiment was terminated when the adult beetles emerged and fresh weight was measured within 12 h after emergence.

### Toxicity of Cry Proteins to Larvae of *P. japonica*

#### Life-table parameter measurement

Neonates of *P. japonica* were individually fed with the pollen/aphid diet containing: (1) Cry1Ac; (2) Cry1Ab; (3) Cry1F; (4) E-64 (positive control); (5) no added toxin (negative control). The Cry proteins were mixed with the pollen at a nominal concentration of 500 µg/g FW of pollen. The concentration of E-64 was 400 µg/g FW of pollen. The diets were provided to the ladybird larvae as described above. Diets were prepared 3 days before initiation of the study and were stored at −20°C until use. Diets were replaced every day to prevent the degradation of the test compounds.

Forty-five individual *P. japonica* larvae were tested for each treatment. Larval development and mortality were recorded twice per day (9∶00 am, 9∶00 pm), and emerging adults were sexed and weighed (within 12 h).

#### Uptake of cry protein by *P. japonica* larvae

Fifty *P. japonica* larvae were fed with the pollen-based diet containing Cry1Ab, Cry1Ac, or Cry1F at 500 µg/g FW of pollen according to the method described above. When the insects reached the fourth instar (just after molting) and pupal stage, 3 samples of insects (3–5 individuals per sample) were collected for each treatment. The insects were frozen at −80°C for later ELISA measurements.

#### Stability and bioactivity of cry protein in food source

The temporal stability and bioactivity of the Cry proteins in the pollen were assessed in three to five subsamples that were collected from fresh pollen and from pollen that had been exposed to *P. japonica* larvae for 2 days. The Cry protein concentrations and bioactivities were determined by ELISA and by a sensitive-insect bioassay (see below).

#### ELISA measurements

The Cry protein concentrations in *P. japonica* samples (3–5 insects per sample, 3 samples per treatment) and in pollen samples (1.0 mg FW of pollen per sample, 3 samples per treatment) were measured by DAS-ELISA using the Cry1Ab, Cry1Ac, and Cry1F detection kits from Agdia (Elkhart, Indiana, USA). Before analyses, all insects were washed in Phosphate-buffered Saline Tween (PBST) to remove any *Bt* toxin from their outer surface. For Cry protein extraction, samples of insects or artificial diets were weighted and mixed with PBST at a ratio of at least 1∶10 to 1∶100 (mg sample:µl buffer) in 2-ml centrifuge tubes. Subsequently after two 3-mm tungsten carbide balls were added into each tube, the samples were macerated for 4 min at 30 Hz in a mixer mill MM400 (Retsch, Haan, Germany) fitted with 24-tube adapters for microreaction tubes. After centrifugation and appropriate dilution of the supernatants, ELISA was performed according to the manufacturer’s instructions. The measured OD values were calibrated to a range of concentrations of Cry1Ab, Cry1Ac, and Cry1F standards made from purified toxin solutions.

#### Sensitive-insect bioassay

Cry protein bioactivity was measured for samples of pollen containing Cry1Ab, Cry1Ac, Cry1F that had been freshly prepared or had been exposed to *P. japonica* larvae for 2 d. Supernatants from the extracts used for the ELISA analysis were appropriately diluted and thoroughly incorporated into the artificial diet for *C. suppressalis* [54]. The final concentration of each Cry protein in the diet was close to its EC50 as described above. Extract of pollen to which no Cry protein was added served as control. The artificial diets were cut into slices and individually placed in Petri dishes (9 cm diameter, 1 cm height) together with a neonate larva of *C. suppressalis*. Subsequently, the Petri dishes were sealed with Parafilm. Thirty replicates were tested for each treatment. After 7 days, the *C. suppressalis* were weighed.

### Data Analysis

The pollen consumption by *P. japonica* at each instar was compared using one-way ANOVA followed by Tukey HSD test. In the experiment evaluating the fitness of *P. japonica* feeding three different diets, Student’s t-tests were performed for comparisons for adult weight and total fecundity. In the experiments with E-64 and Cry proteins, statistical comparisons were made between each treatment and the control (pure diet) using Dunnett’s test for the parameter of adult weight because the assumptions for parametric analysis (normal distribution of residues and homogeneity of error variances) were met. The assumptions for parametric analyses were not met for the pupation and eclosion rates, and insect development time, so such data were analyzed by *χ^2^*-test and Mann-Whitney U-test. The Bonferroni correction was applied for these statistical tests to correct for multiple pair-wise comparisons. The effect of dietary treatments on *P. japonica* survival was analyzed with the Kaplan-Meier procedure and Logrank test.

In addition, Student’s t-tests were used to compare Cry protein concentrations in the fresh pollen vs. pollen exposed to *P. japonica* larvae for 2 days, and one-way ANOVA followed by Tukey HSD tests were carried out to compare the 7-d larval weight of *C. suppressalis* that was fed with artificial diets containing the extracts from pure fresh pollen or extracts from pollen containing Cry proteins.

All above statistical analyses were conducted using the software package SPSS (version 13 for windows, 2004).
